# Predicting Local Dengue Transmission in Guangzhou, China, through the Influence of Imported Cases, Mosquito Density and Climate Variability

**DOI:** 10.1371/journal.pone.0102755

**Published:** 2014-07-14

**Authors:** Shaowei Sang, Wenwu Yin, Peng Bi, Honglong Zhang, Chenggang Wang, Xiaobo Liu, Bin Chen, Weizhong Yang, Qiyong Liu

**Affiliations:** 1 State Key Laboratory for Infectious Disease Prevention and Control, National Institute for Communicable Disease Control and Prevention, Chinese Center for Disease Control and Prevention, Changping, Beijing, China; 2 Key Laboratory of Surveillance and Early-Warning on Infectious Disease, Chinese Center for Disease Control and Prevention, Beijing, China; 3 School of Population Health, University of Adelaide, Adelaide, South Australia, Australia; 4 Department of Preventive Medicine, College of Basic Medical Sciences, Shandong University of Traditional Chinese Medicine, Jinan, Shandong, China; 5 Xiamen Entry-Exit Inspection and Quarantine Bureau, Xiamen, China; 6 WHO Collaborating Centre for Vector Surveillance and Management, Changping, Beijing, China; 7 Centre for Environment and Population Health, Nathan Campus, Griffith University, Nathan, Queensland, Australia; 8 Shandong University Climate Change and Health Center, Jinan, China; 9 Collaborative Innovation Center for Diagnosis and Treatment of Infectious Diseases, Hangzhou, China; Duke-National University of Singapore Graduate Medical School, Singapore

## Abstract

**Introduction:**

Each year there are approximately 390 million dengue infections worldwide. Weather variables have a significant impact on the transmission of Dengue Fever (DF), a mosquito borne viral disease. DF in mainland China is characterized as an imported disease. Hence it is necessary to explore the roles of imported cases, mosquito density and climate variability in dengue transmission in China. The study was to identify the relationship between dengue occurrence and possible risk factors and to develop a predicting model for dengue’s control and prevention purpose.

**Methodology and Principal Findings:**

Three traditional suburbs and one district with an international airport in Guangzhou city were selected as the study areas. Autocorrelation and cross-correlation analysis were used to perform univariate analysis to identify possible risk factors, with relevant lagged effects, associated with local dengue cases. Principal component analysis (PCA) was applied to extract principal components and PCA score was used to represent the original variables to reduce multi-collinearity. Combining the univariate analysis and prior knowledge, time-series Poisson regression analysis was conducted to quantify the relationship between weather variables, Breteau Index, imported DF cases and the local dengue transmission in Guangzhou, China. The goodness-of-fit of the constructed model was determined by pseudo-*R^2^*, Akaike information criterion (AIC) and residual test. There were a total of 707 notified local DF cases from March 2006 to December 2012, with a seasonal distribution from August to November. There were a total of 65 notified imported DF cases from 20 countries, with forty-six cases (70.8%) imported from Southeast Asia. The model showed that local DF cases were positively associated with mosquito density, imported cases, temperature, precipitation, vapour pressure and minimum relative humidity, whilst being negatively associated with air pressure, with different time lags.

**Conclusions:**

Imported DF cases and mosquito density play a critical role in local DF transmission, together with weather variables. The establishment of an early warning system, using existing surveillance datasets will help to control and prevent dengue in Guangzhou, China.

## Introduction

The potential Dengue Fever transmission to areas currently free of the disease is a significant concern from the dengue control perspective [1]. Dengue is mostly endemic in tropical and sub-tropical countries, of which most have attractive tourist destinations. The rise of international travel plays an important role in the global spread of dengue. Travelers infected with dengue virus during their trip returning home may place the local population at risk wherever *Ae. aegypti* and *Ae. albopictus* are present. Due to the establishment of *Ae. albopictus* in south-east France, Nice reported local dengue transmission for the first time in 2010 [2]. The third largest Dengue Fever outbreak with 1,010 cases in Guangdong Province of China in 2006 was confirmed being caused by imported cases from Southeast Asia [3]. The first report of DF outbreak in Dongguan, Guangdong Province was also caused by imported cases from Southeast Asia [4]. Furthermore, an outbreak caused by re-emergence of dengue virus 4 in 2010, a serotype of dengue which disappeared in China for 20 years, was also caused by an imported case from Thailand [5].

Although a few studies have explored the impact of climate variability on DF transmission, the strength of the association varied with time and locations in Asia-Pacific region [6]. A study conducted in Guangdong Province, China, for example, showed that the daily vapour pressure, mean and minimum temperatures were positively associated with DF transmission but maximum temperature and Southern Oscillation Index (SOI) were negatively associated [7]. Another study conducted in Guangzhou city, China showed that DF transmission was positively associated with minimum temperature at 1 month lag and average relative humidity at 0 to 1 month lag, while negatively associated with wind velocity and minimum temperature in the same month and rainfall with 2 month lagged effects [8]. Lu *et al.* found that minimum temperature and minimum humidity at one month lag were positively associated with DF incidence, while wind velocity was inversely associated with DF incidence of the same month [9].

There was no dengue cases reported in China over the period of 1949 to 1977. The first outbreak of DF in mainland China was identified in Guangdong Province in 1978. The DF epidemic has since then gradually spread from Guangdong, Hainan, Guangxi Provinces in the southern coastal regions to other regions, including Fujian, Zhejiang and Yunnan Provinces [10]. DF outbreaks have occurred in different scales in China since 1978. There were 655,324 cases and 610 deaths being notified over the period 1978–2008 [10]. In recent years, Guangdong Province has had the highest incidence of dengue in China with cases reported every year since 1997 [7], [11]. Still being characterized as an imported disease, DF in mainland China has not been confirmed to be endemic [12].

Global trade, increasing travel with population movement, crowded urban living conditions, global warming, virus evolution and ineffective vector-control strategies are increasing the risk of spreading dengue transmission in the world [13], [14]. While risk factors associated with dengue occurrence in China are still not clear, and most of the studies conducted in China mainly used weather variables to predict DF risk [7], [8], [9]. It is probably better to consider other potential risk factors in the prediction including mosquito density. The aim of this project is to identify risk factors for dengue outbreak and transmission, and to predict possible further outbreak. Furthermore the analytic results will be useful to develop a dengue early warning system and will provide important evidence for dengue control policy implications and public health intervention.

## Materials and Methods

### Ethics Statement

Ethical approval for this project was obtained by Chinese Center for Disease Control and Prevention Ethical Review Committee (No. 201214) and patient data used in the study were de-identified.

### Study areas

Guangdong Province is in southeast China. Guangzhou city is the capital of Guangdong Province, its precise location being 112°57′E to 114°03′E and 22°26′N to 23°56′N. Guangzhou city is situated in the central south of Guangdong Province, bordering the Pearl River Delta. Guangzhou has a subtropical monsoon climate with hot and humid Summer; mild, dry and sunny Winter. The annual mean temperature is 22°C. The annual accumulate precipitation is 1,736 mm.

**Figure 1:**
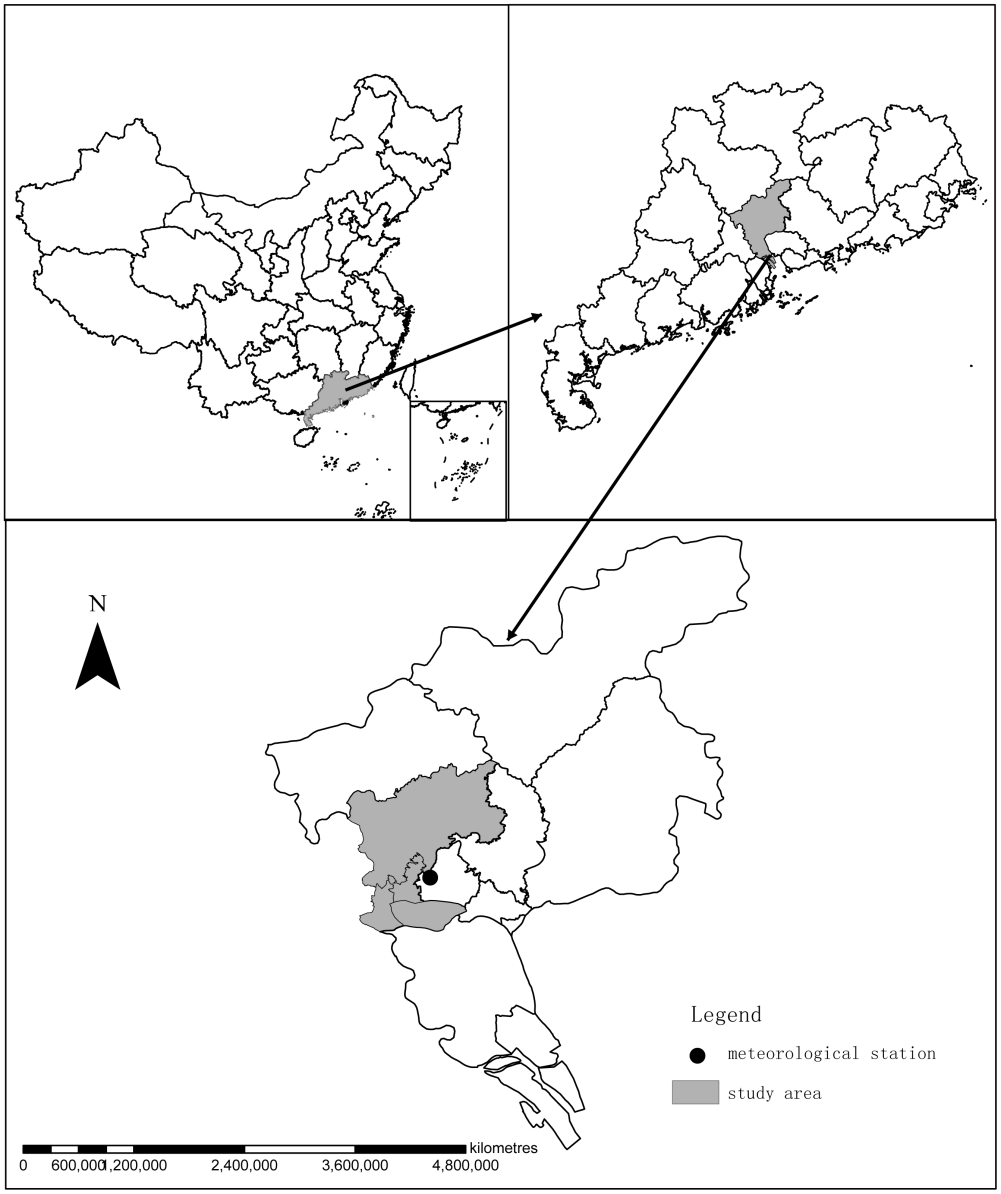
Study areas in Guangzhou city, China.

Guangzhou city consists of 10 districts and 2 satellite cities. Four districts named *Baiyun district, Yuexiu district, Liwan district, Haizhu district* were chosen as the study areas (Figure 1), including three traditional suburbs and a district with Buiyun International Airport. The area of the four districts is 979.09 km^2^ with population 5.81million in 2013.

### Data collection

#### Dengue data

Records of dengue cases between March 2006 and December 2012 in the selected study regions were obtained from the China National Notifiable Disease Surveillance System. DF is a notifiable disease, and all cases of DF are diagnosed according to the China National diagnostic criteria for Dengue Fever (DF) (WS216–2008) [4], [15]. The information of dengue cases included age, sex, occupation, date of onset, whether the diagnosis was clinical or confirmed by laboratory test, local case or not and source country if the case was imported. Routine case notification is performed by hospitals as required by law. When DF outbreaks occur in the community possible DF cases are also detected by active field investigation performed by health professionals. Therefore, dengue surveillance involves both passive and active case detection.

The criteria of imported cases included (1) residency or traveling experience of in a DF endemic country or Taiwan of China and bitten by a mosquito within 15 days before symptoms appeared; or (2) the gene sequence of the virus isolated from the case is highly homologous with that reported by the country to/from which the patient had travelled. A local case is defined according to the absence of evidence for the case being imported [16].

#### Weather data

The monthly weather data were obtained from the China Meteorological Data Sharing Service System http://cdc.cma.gov.cn/home.do. The meteorological variables included extreme minimum temperature (ExMinT), mean minimum temperature (MinT), mean temperature (MT), mean maximum temperature (MaxT), extreme maximum temperature (ExMaxT), mean relativehumidity (RH), mean minimum relative humidity (MinRH), mean vapor pressure (MVP), extreme minimum air pressure (ExMinAP), extreme maximum air pressure (ExMaxAP), mean air pressure (MAP), accumulative precipitation, maximum daily precipitation (MaxDP), extreme wind velocity (ExWV), mean wind velocity (MWV), and mean maximum wind velocity (MaxWV). There are two meteorological stations in Guangzhou city (Figure 1), and the climate data used were monitored by meteorological station A. There was no data missing over the study period from March 2006 to December 2012.

#### Entomologic data

*Ae. Albopictus* is the sole transmission vector in Guangzhou city [17]. Three larval indices are commonly used to assess the density levels of *Ae. aegypti* and *Ae. albopictus*: House Index (HI), Container Index (CI) and Breteau Index (BI). As HI does not consider the number of positive containers per house and the CI only provides information on the proportion of water-holding containers that are positive, the BI is considered the best single index for *Aedes* density surveillance, which has been used in this study. Larval density was monitored in residential areas, parks, construction sites, and hospitals in every district (county). Over 50 households were sampled to conducted surveillance in residential areas monthly. In other places such as parks, construction sites, the survey was conducted every 10 meters (per household equivalent) until the 50 houses equivalent targets were achieved. All types of water containers were thoroughly checked for *Ae. Albopictus* breeding and if found positive the larvae would be picked up for the confirmation of *Ae. Albopictus* larvae. BI equals to number of positive containers per 100 houses inspected. The mosquito surveillance was conducted monthly over the study period with no data missing.

#### Population data

The population data over the study period for every district was retrieved from the Guangdong Statistical Yearbook.

### Statistical analysis

#### Autocorrelation analysis and cross-correlation analysis

Autocorrelation analysis was conducted to explore whether the monthly local DF cases were affected by previous local cases, by using the Ljung-Box Q test, autocorrelation coefficient (AC) and partial autocorrelation coefficient (PAC). Cross-correlation analysis was used to detect the correlation between monthly local DF cases and climatic factors, BI, imported cases with a lag time of 7 months. The cross-correlation terms were retained in the model if the absolute value of cross-correlation coefficient (CCF) was two times larger than the standard error (SE) [18].

#### Principal component analysis (PCA)

The variables associated with local cases determined by cross-correlation analysis were correlated with each other. In order to reduce the multi-collinearity when putting all the correlated variables in model, PCA was conducted first to extract principal components to represent all the correlated variables. The number of the principal components depended on Initial Eigenvalue (>1) or accumulated contribution rate (>80%).

#### Time-series Poisson analysis

Based on the univariate analysis and prior knowledge, a time-series Poisson model was used to examine the relationship between local DF cases and the independent variables. In order to control the influence of long-term trend, “year” was put into the model as an independent variable [19], [20]. Potential seasonality was controlled by adding an ordinal categorical seasonal variable: 0-Autumn (September to November), 1-Summer (June to August), 2-Spring (March to May), 3-Winter (December to February) [19] (We also treated “Season” as dummy variables to recalculate the model and the results were in Results S1 ). The interaction effect may exist between variables. The interaction term was calculated with multiplication. The logarithmic population (logpop) variable was the offset. The goodness-of-fit of the constructed models was determined by residual test, pseudo-*R^2^* [20] and Akaike information criterion (AIC) [21]. The data from March 2006 to December 2011 were used to develop the model and the data from January 2012 to December 2012 were employed to validate the model. All the time-series variables graphs were finished by Microsoft office excel 2007. The autocorrelation, cross-correlation analysis and PCA analysis were finished by SPSS software (version 16.0). Stata software (version 11.0) was used to perform time-series Poisson regression analysis.

## Results

### Description of local DF cases in the four districts, 2006–2012

**Figure 2:**
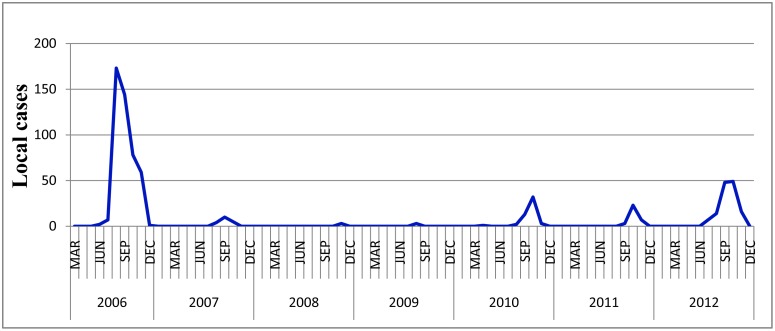
Temporal distribution of local DF cases, 2006–2012.

There were total 707 notified local DF cases over the study period. The local DF mainly occurred from August to November, late Summer and Autumn, with a large outbreak in 2006 (Figure 2). The average incidence was 28.27 per million, with 132.0, 5.36, 0.837, 0.830, 13.9, 8.93, 36.0 per million each year over the study period.

### Description of imported cases in the four districts, 2006–2012

**Figure 3:**
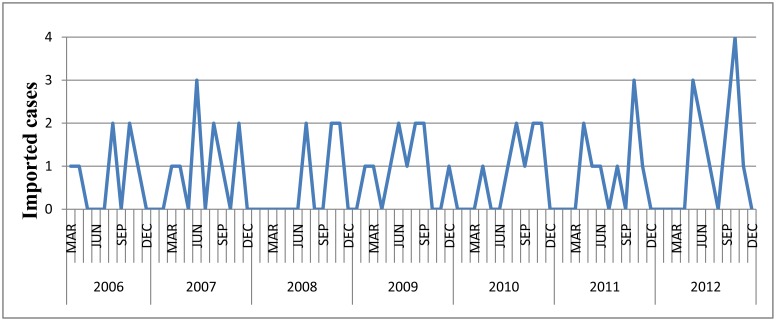
Temporal distribution of imported DF cases, 2006–2012.

**Figure 4:**
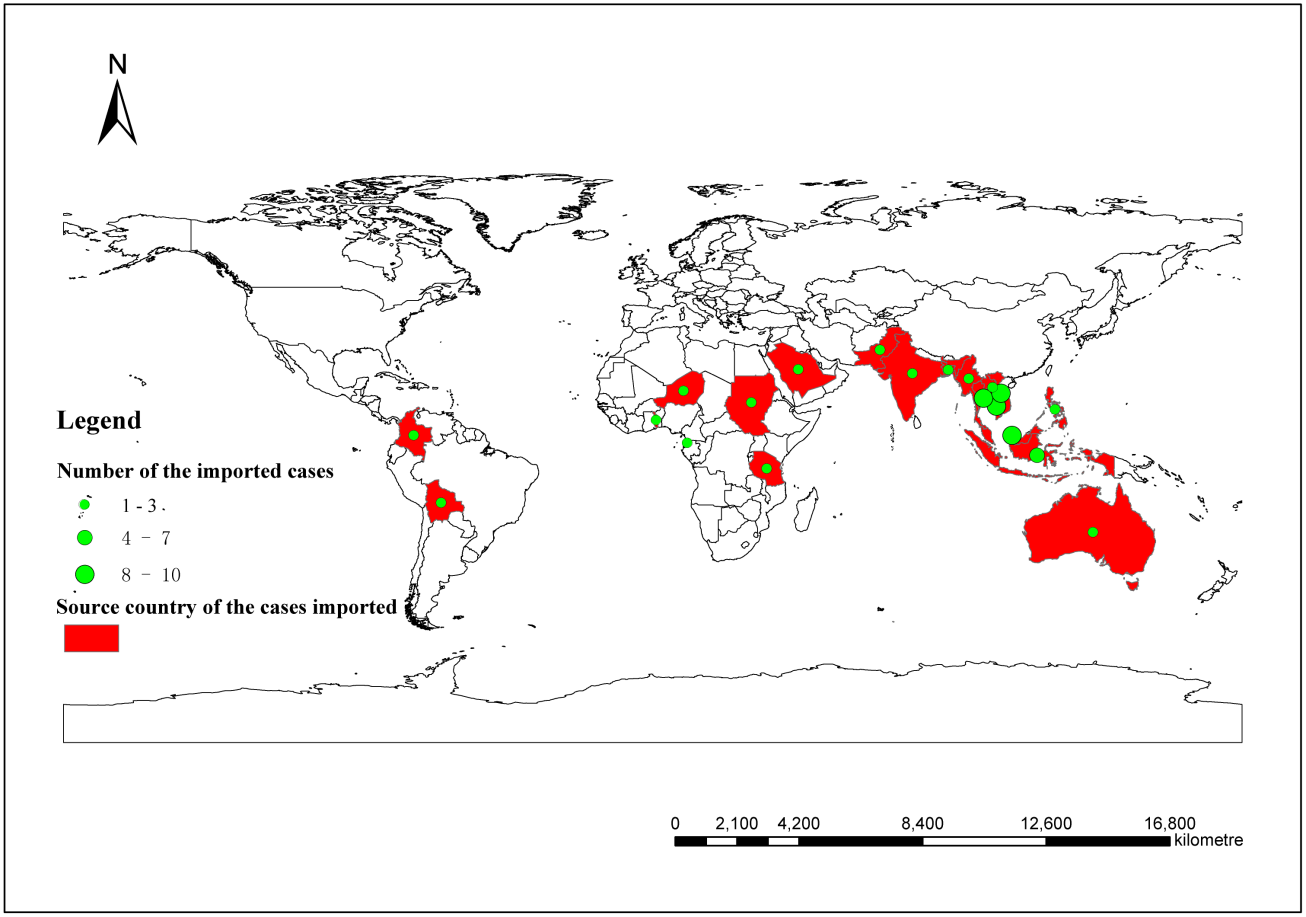
The geographical distribution of imported DF cases worldwide.

There were total 65 notified imported DF cases over the study period, which were notified almost every month (Figure 3). The cases were imported from 20 countries including Thailand, Vietnam, Cambodia, Indonesia, Malaysia, Bangladesh, Australia, Bolivia, Colombia, Niger, Pakistan, Philippines, Saudi Arabia, Sudan, Tanzania, India, Togo, Laos, Myanmar and Guinea. Forty-six cases (70.8%) were imported from Southeast Asia with Thailand 10, Vietnam 8, Cambodia 10, Indonesia 7, Malaysia 8 cases, Laos 1 and Myanmar 2 (Figure 4).

### Temporal variation in BI and weather variables

**Figure 5:**
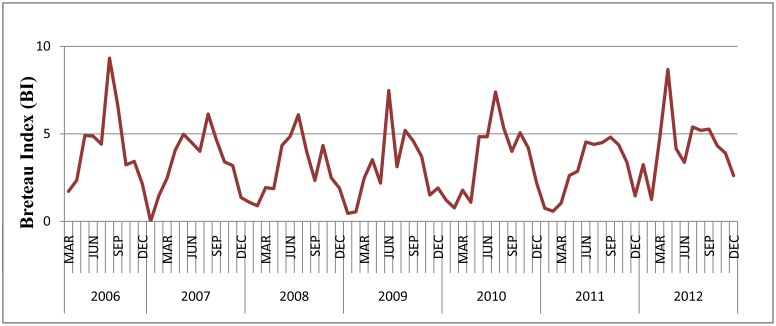
The dynamic variation of *Ae. albopictus* density in the study areas, 2006–2012.

The density of *Ae. albopictus* showed dynamic and periodic variation. The BI over 5 usually appeared in Summer and early Autumn in Guangzhou (Figure 5).

Seasonal variation was observed in air pressure variables, temperature variables and MVP variable. Accumulative precipitation, MaxDP and MinRH had a higher inter-annual variation. The variation of relative humidity and wind velocity variables was minor (data not shown but available on request).

### Auto-correlation analysis of monthly local DF cases

The auto-correlation analysis showed the autocorrelation coefficient delayed at lags 1–2, associated with a partial autocorrelation coefficient cutoff at lag 1, suggesting a non-seasonal 1 lag autocorrelation, which indicated that the number of dengue local cases in the current month was correlated to the number of cases occurring in the previous month.

### Cross-correlation analysis between monthly local DF cases and independent variables

Cross-correlation analysis showed that monthly local DF cases were negatively associated with air pressure variables, while positively correlated with temperature variables, vapour pressure variables, precipitation variables and BI variables with different lags of month, but no significant relationship with imported cases (Table S1).

### The extraction of principal components

The weather variables and BI were correlated with each other (Table S2). These correlated variables were analyzed with PCA method and four principal components (FAC_1_, FAC_2_, FAC_3_, FAC_4_) (Formula S1) were extracted, with the cumulative contribution rate 88.9% (Table S3). The eigenvectors of every principal component on each climate variables and BI can be seen from Table S4.

### Time-series Poisson model construction

The variables put into the model depended on the univariate and empirical analyses. The number of local cases was used as dependent variable. The autocorrelated term (local cases with one month lag named Local_1_), FAC_1_, FAC_2_, FAC_3_ and FAC_4_ were screened by univariate analysis. We also put imported cases (Imp) with different lags as independent variables according to literature, although Imp variable was not correlated with local cases with univariate analysis. Interaction effects were also investigated.

**Figure 6:**
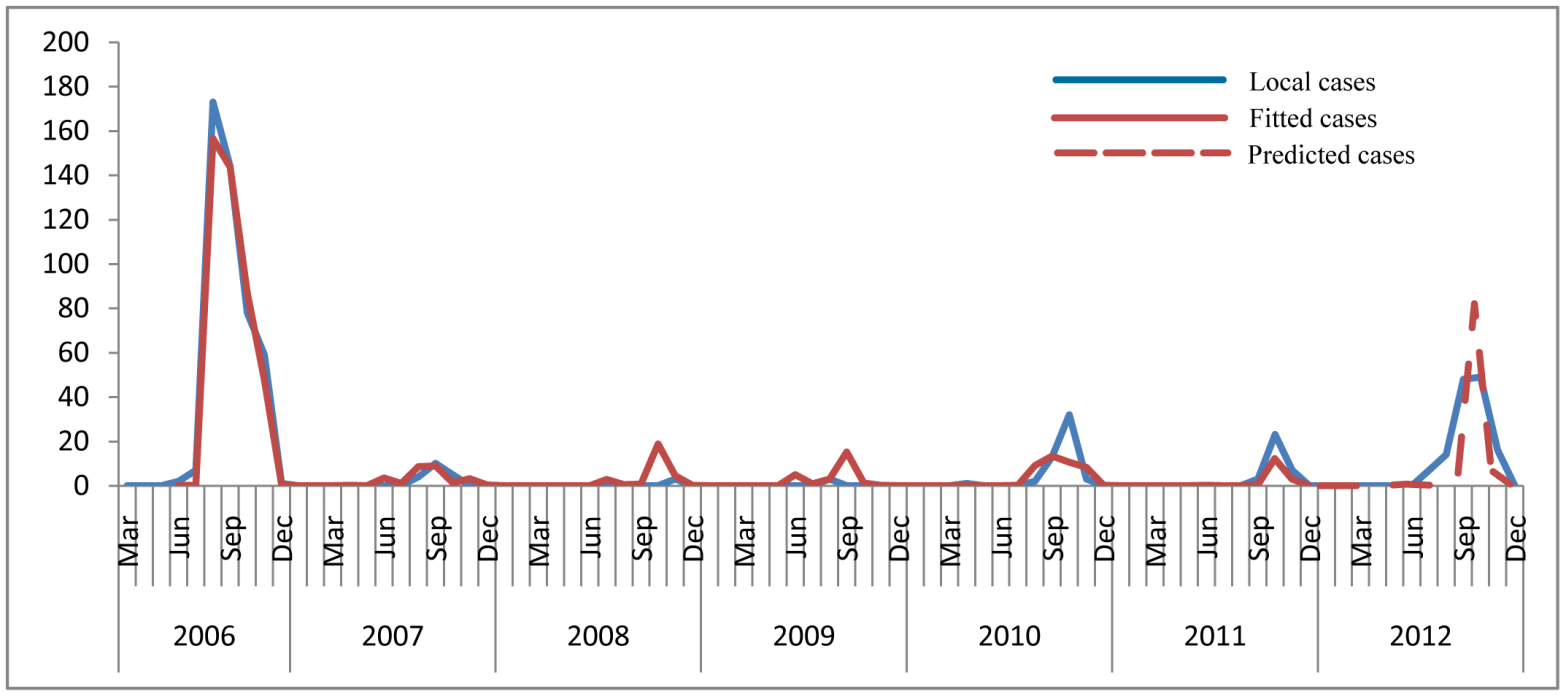
The time series of fitted DF cases and the local DF cases.

Based on the parameters of AIC, Pseudo *R^2^* and the residual test, the finial predictors included in the model are presented in Table 1. Pseudo *R^2^* = 0.879. The correlation coefficient was 0.984 between local DF cases and the fitted DF cases. The residual test showed that the residuals were not correlated with different lags (*p>0.05*) (Table 2), which indicated that the information of these variables was extracted sufficiently. The time series of fitted DF cases coincided with local DF cases well in the non-epidemic period and epidemic period (Figure 6). The best-fit model was as follows:
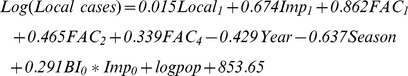



**Table 1:** Variables used to build the time-series Poisson model.

	Coef.	Std. Err.	z	P>z	[95% Conf. Interval]	
**Local_1_**	0.015	0.001	10.310	0.000	0.013	0.018
**Imp_1_**	0.674	0.085	7.900	0.000	0.507	0.841
**FAC_1_**	0.862	0.246	3.510	0.000	0.381	1.344
**FAC_2_**	0.465	0.122	3.820	0.000	0.227	0.703
**FAC_4_**	0.339	0.060	5.640	0.000	0.221	0.456
**Year**	−0.429	0.045	−9.550	0.000	−0.517	−0.341
**Season**	−0.637	0.244	−2.610	0.009	−1.115	−0.159
BI_0_*Imp_0_	0.291	0.021	14.060	0.000	0.251	0.332
**Constant**	853.650	90.294	9.450	0.000	676.677	1030.623
**logpop**	(offset)					

**Table 2:** Residual correlation test.

LAG	AC	PAC	Q	Prob>Q
**1**	0.024	0.024	0.040	0.841
**2**	−0.124	−0.126	1.132	0.568
**3**	0.156	0.162	2.890	0.409
**4**	0.035	0.023	2.979	0.561
**5**	0.019	0.061	3.006	0.699
**6**	0.012	−0.007	3.017	0.807
**7**	−0.027	−0.031	3.072	0.878
**8**	0.063	0.062	3.380	0.908
**9**	−0.017	−0.032	3.402	0.946

Note: AC was autocorrelation coefficient and PAC was partial autocorrelation coefficient. Q was the value of Ljung-Box Q test. P>0.05 meant that residuals were not correlated.

The final model was:
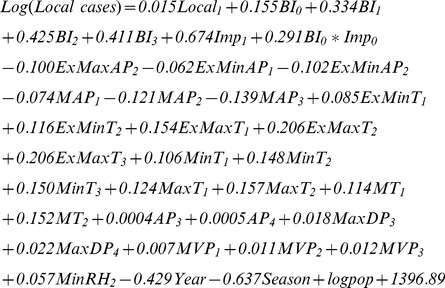



From the model we could find that “Season” variable was significant, which indicated that the occurrence of local cases had a seasonality. After controlling the impact of seasonality and long-term trend, the model showed local cases were positively associated with one lag month of local cases and imported cases, positively associated with *Ae. albopictus* density with lags of 0–3 month, negatively associated with ExMaxAP with 2 month lags, ExMinAP with 1–2 month lags, MAP with 1–3 month lags, positively associated with ExMinT with 1–2 month lags, ExMaxT with 1–3 month lags, MinT with 1–3 month lags, MaxT with 1–2 month lags, MT with 1–2 month lags, positively associated with AP with 3–4 month lags, MaxDP with 3–4 month lags, positively associated with MVP with 1–3 month lags, positively associated with MinRH with 2 month lags. The local cases were also positively with BI_0_*Imp_0_, indicating that the existence of imported cases increased the effect of *Ae. albopictus* on dengue transmission in the current month.

## Discussion

The results showed that imported cases, mosquito density and weather variables could be used to predict local dengue transmission. To our best knowledge this is the first such study incorporating both imported cases and mosquito density in the predictive model, together with weather variables.

With our world becoming increasingly globalized, dengue has become the second most common cause of diagnosed disease after foreign travel, following malaria [22]. Our study showed that forty-six cases (70.8%) in Guangzhou were imported from Southeast Asia. Several dengue outbreaks in China have been confirmed, through phylogenetic and epidemiological analysis, that the outbreaks were caused by imported cases from Southeast Asia [3], [4], [5], [23], Indian subcontinent or western Asia [15], indicating the importance to enhance border quarantine service. Furthermore, Shang *et al* demonstrated that imported DF cases served as an initial facilitator or spark for possible dengue transmission in an area with lower population immunity and the occurrence of local DF was significantly correlated with temporally-lagged imported dengue cases (2–14 weeks) in Taiwan [24]. Our study showed that local DF was associated with imported DF cases with 0–1 month lagged effect. All of these demonstrated that imported dengue cases could lead to local dengue transmission and may have a quantitative relationship with local cases up to next month in the context of suitable climatic conditions and necessary mosquito density.

Although only adult female *Aedes* mosquitoes are directly involved in dengue transmission, a number of indices have been used to monitor *Aedes* density in terms of dengue transmission. The Breteau index (BI) was the most widely used index because of its high correlation with adult mosquito density [25]. One case-control study showed that before or after DF outbreak, over 6% of blocks without dengue cases had BI >4, with a maximum BI of 50[26], which indicated that the relationship between mosquito density and dengue infection was dynamic and complex. Some studies found there was no relationship between BI and dengue transmission [27], [28], whereas others demonstrated that mosquito density was correlated with dengue outbreak. Moore *et al* found that the peak incidence of confirmed dengue infections followed the peak larval density by approximately one month in Puerto Rico [29]. Pontes *et al* demonstrated that mosquito density was correlated with dengue outbreak in epidemic areas and each outbreak followed a period of relaxed monitoring and source-reduction activity with a consequent increase in the abundance of vector mosquitoes [30]. Pham *et al* also demonstrated that dengue cases were positively associated with the BI [31]. Morrison and Scott determined that traditional indices for *Ae. aegypti* density were correlated with the prevalence of human dengue infections, but were at best weakly correlated with the incidence in a large, cohort-based prospective study [32], which could be due to other factors such as herd immunity, mosquito-human interaction, virus strain and daily mosquito survival rate influenced the relationship between *Aedes* density and dengue transmission [33]. Our study indicated that local dengue cases were positively associated with temporally-lagged BI (0–3 months). Dengue in mainland China is still characterized as an imported disease and has not yet been confirmed to be endemic. The immunity level against dengue is low within the Chinese population. As the mosquito density continues to grow, dengue virus transmission becomes easier and the risk of DF outbreak is likely to increase.

Our study also indicated that mosquito density and imported cases had an interaction effect and imported cases positively modified the relationship between mosquito density and local dengue occurrence in the current month, which indicated that the value of imported cases increased the effect of mosquito density on local dengue transmission.

The results showed that local cases were positively associated with temperature variables with different lagged effects. Higher temperatures may reduce the mosquito development time [34], which could bring about more mosquitoes in a shorter time. Repeated feeding on human blood can increase the efficiency of virus transmission. The frequency of blood feeding is influenced both indirectly and directly by ambient temperature. Smaller mosquitoes feed more often than larger ones [35], while higher temperatures could augment immature development resulting in smaller mosquitoes [36]. Temperature also influenced the interaction between dengue virus and *Ae. albopictus*. Virus spread throughout the body of *Ae. albopictus*, a pre-requisite for transmission, was reduced when the immature stages were reared in cool conditions [37]. Transmission models showed that the reduced mosquito life expectancy offset the increased biting rate and virus propagation beyond 40°C[38]. However, temperatures above 30°C may have minimal impact on the *Aede* mosquito in the field, as it may avoid excessive daytime heat by resting in cooler and more obscure indoor locations [39]. Wu *et al* found that with every 1°C increase of monthly average temperature may bring about 1.95 times of the risk for dengue transmission [40]. Temperature is closely related with the extrinsic incubation period (EIP). Laboratory studies have proven that the EIP decreased with increasing temperatures [41], [42]. Furthermore, using censored Bayesian time-to-event model Miranda *et al* demonstrated that EIP decreased from 15 days at 25°C to 6.5 days at 30°C [43]. All these results supported our findings. In terms of the interaction between climate and vector, a suitable temperature may create an excellent micro-environment for vector to have a faster growth and development, and so is the rainfall. Furthermore, weather may change human behaviour, which may increase the contact opportunities between the vector and human beings.

After the long-term trend and seasonality were controlled in the model, the monthly mean minimum relative humidity (MinRH_2_) was positively correlated with local DF cases, indicating that the probability of dengue occurrence is larger with higher mean minimum relative humidity. Lu *et al* also showed that minimum humidity would be helpful to predict DF cases [9].

Our results showed that local DF cases were positively associated with precipitation variables with 3 to 4 months when the peak of dengue cases coincided with the peak precipitation variables. *Ae. albopictus* can breed in both domestic and peri-domestic containers, rainfall can create more breeding sites, which may lead to an increase in the number of mosquitoes. Previous studies also showed that dengue transmissions were positively associated with rainfall [31], [44]. Rainfall might indirectly increase the biting rate of mosquito. Humans often stay indoors when it rains, which increases the contact between humans and mosquitoes.

Vapour pressure, a combined variable of humidity and temperature was associated with a high historical incidence of dengue, contributing to an elevated local *Aedes aegypti* infestation rate [45]. Hales *et al* found that vapour pressure were associated with dengue transmission and projected the world distribution of dengue on the basis of climate projections on vapour pressure by 2055 and 2085 [46]. The positive result between vapour pressure and dengue transmission was also found in our study.

There has been minimal research of the role of atmospheric pressure on dengue in scientific literatures. Our study showed that local dengue cases were negatively associated with atmospheric pressure, while a study in Brazil found that dengue cases were positively associated with atmospheric pressure in the early-year, whilst being negatively associated with the disease in mid- and late-year [47].

Dengue is not endemic in Guangzhou and the situation for dengue transmission is more complicated in China. Our study demonstrated that imported cases, mosquito density and climate variability played a great role in dengue transmission. Dengue serotype or genotype also plays a critical role in dengue outbreak in China. However, we can not predict or quantify dengue serotype or genotype, so it is unavailable to use this kind information to predict dengue transmission in China. Predictive model showed that the mosquito density and imported cases variables at the current month may play an important role in the local dengue transmission, suggesting that the mosquito may bite the imported cases and complete the EIP, and then infect local cases in one month time when other environmental conditions are suitable [43]. In the public heath practice, it will be ideal to have weekly datasets so the projections could be more accurate, however such information is not available.

Based on the results from this study and findings from other published literatures, it is reasonable to construct a dengue early warning system incorporating imported cases, mosquito density and climate variability in the predictive model. Furthermore, the results from this study may provide important policy implications and preventative guidelines for local health authorities for their prevention strategies and measurements implementations. The results may also provide useful information to Asia and Pacific region for their dengue control and prevention.

### Strengths and limitations

This study has been the first to evaluate the relationship between local dengue transmission and imported cases, mosquito density and climate variability. Imported cases play a critical role in dengue transmission in China, it is therefore important to take this variable into consideration in the establishment predictive model and implementation of intervention program.

This study also has limitations. Some factors influenced the relationship between *Aedes* density and dengue transmission, such as herd immunity, mosquito-human interaction, virus strain and mosquito daily survival rate [33]. In our study we were unable to evaluate the mosquito-human contact rate and mosquito survival rate. As to the relationship between mosquito density and dengue transmission, the result was based on a hypothesis that mosquito density was positively associated with biting rate. Complicated stochastic simulation models have been developed to describe the daily dynamics of dengue virus transmission, which take into account the majority of factors known to influence dengue epidemiology [48]. We may explore such work in future.

### Conclusions

Local dengue occurrence in Guangzhou has a seasonal distribution. Imported dengue cases and mosquito density play an important role in local case occurrence. Besides mosquito density, further attention should be paid to the imported cases in China. Lower local air pressure, higher temperature, more precipitation and higher minimum relative humidity will increase the risk of local DF transmission in the context of imported dengue cases and mosquito density.

## Supporting Information

Table S1 **Cross-correlation analysis between local DF cases and BI, imported cases, weather variables.**
(XLSX)

Table S2 **Correlation Matrix between climate variables and mosquito density with different lags of month.**
(XLSX)

Table S3 **Components extraction and total variance explained.**
(XLSX)

Table S4 **Eigenvectors of FAC on every original variables.**
(XLSX)

Formula S1 **The formulas of the four principal components (FAC 1–4).**
(DOCX)

Results S1 **The results of predicting model when “Season” variable replaced with three dummy variables (Season_summer_, Season_spring_ and Season_winter_).**
(ZIP)
