# Supplementary material for: Predicting Local Dengue Transmission in Guangzhou, China, through the Influence of Imported Cases, Mosquito Density and Climate Variability
Source: PLoS One. 2014 Jul 14;9(7):e102755. doi: 10.1371/journal.pone.0102755 (PMC4097061; doi:10.1371/journal.pone.0102755)
Supplement: Formula S1 — The formulas of the four principal components (FAC 1–4). (DOCX) [file pone.0102755.s005.docx]

**

**

**

**

***formula (S1)***
