# Supplementary material for: Predicting Local Dengue Transmission in Guangzhou, China, through the Influence of Imported Cases, Mosquito Density and Climate Variability
Source: PLoS One. 2014 Jul 14;9(7):e102755. doi: 10.1371/journal.pone.0102755 (PMC4097061; doi:10.1371/journal.pone.0102755)
Supplement: Results S1 — The results of predicting model when “Season” variable replaced with three dummy variables (Seasonsummer, Seasonspring and Seasonwinter). (ZIP) [file pone.0102755.s006.zip › Results S1.docx]

**The results of “Season” variable treated as dummy variables were as follows:**

Based on the parameters of Pseudo *R^2^* and the residual test, the finial predictors included in the model were presented in Table S1. Pseudo *R^2^*=0.879. The correlation coefficient was 0.984 between local DF cases and the fitted DF cases. The residual test showed that the residuals were not correlated with different lags (*p>0.05*) (Table S2).

The model with principal components was as follows:

The final model was as follows:

Figure S1. The time series of fitted DF cases and the local DF cases.

Table S1. Variables used to build the time-series Poisson model

|  | Coef. | Std. Err. | z | P>z | [95% Conf. Interval] | |
| --- | --- | --- | --- | --- | --- | --- |
| Local_1_ | 0.015 | 0.002 | 10.270 | 0.000 | 0.013 | 0.018 |
| Imp_1_ | 0.685 | 0.087 | 7.900 | 0.000 | 0.515 | 0.855 |
| FAC_1_ | 0.936 | 0.275 | 3.410 | 0.001 | 0.398 | 1.474 |
| FAC_2_ | 0.498 | 0.150 | 3.330 | 0.001 | 0.205 | 0.792 |
| FAC_4_ | 0.328 | 0.064 | 5.130 | 0.000 | 0.202 | 0.453 |
| Year | -0.427 | 0.046 | -9.190 | 0.000 | -0.518 | -0.336 |
| Season_summer_ | -0.581 | 0.364 | -1.600 | 0.110 | -1.294 | 0.132 |
| Season_spring_ | -0.248 | 1.266 | -0.200 | 0.845 | -2.730 | 2.233 |
| Season_winter_ | -2.081 | 1.036 | -2.010 | 0.045 | -4.111 | -0.051 |
| BI_0_*Imp_0_ | 0.292 | 0.023 | 12.910 | 0.000 | 0.248 | 0.337 |
| Constant | 850.161 | 93.460 | 9.100 | 0.000 | 666.984 | 1033.339 |
| logpop | (offset) |  |  |  |  |  |

Table S2. Residual correlation test

| LAG | AC | PAC | Q | Prob>Q |
| --- | --- | --- | --- | --- |
| 1 | 0.055 | 0.055 | 0.211 | 0.646 |
| 2 | -0.197 | -0.202 | 2.971 | 0.226 |
| 3 | 0.205 | 0.239 | 6.017 | 0.111 |
| 4 | 0.021 | -0.067 | 6.048 | 0.196 |
| 5 | 0.016 | 0.110 | 6.066 | 0.300 |
| 6 | 0.007 | -0.039 | 6.070 | 0.415 |
| 7 | -0.028 | -0.007 | 6.128 | 0.525 |
| 8 | 0.014 | 0.008 | 6.144 | 0.631 |
| 9 | -0.008 | -0.027 | 6.149 | 0.725 |

Note: AC was autocorrelation coefficient and PAC was partial autocorrelation coefficient. Q was the value of Ljung-Box Q test. P>0.05 meant that residuals were not correlated.
