# Supplementary figures and images for: Predicting Local Dengue Transmission in Guangzhou, China, through the Influence of Imported Cases, Mosquito Density and Climate Variability
Source: PLoS One. 2014 Jul 14;9(7):e102755. doi: 10.1371/journal.pone.0102755 (PMC4097061; doi:10.1371/journal.pone.0102755)

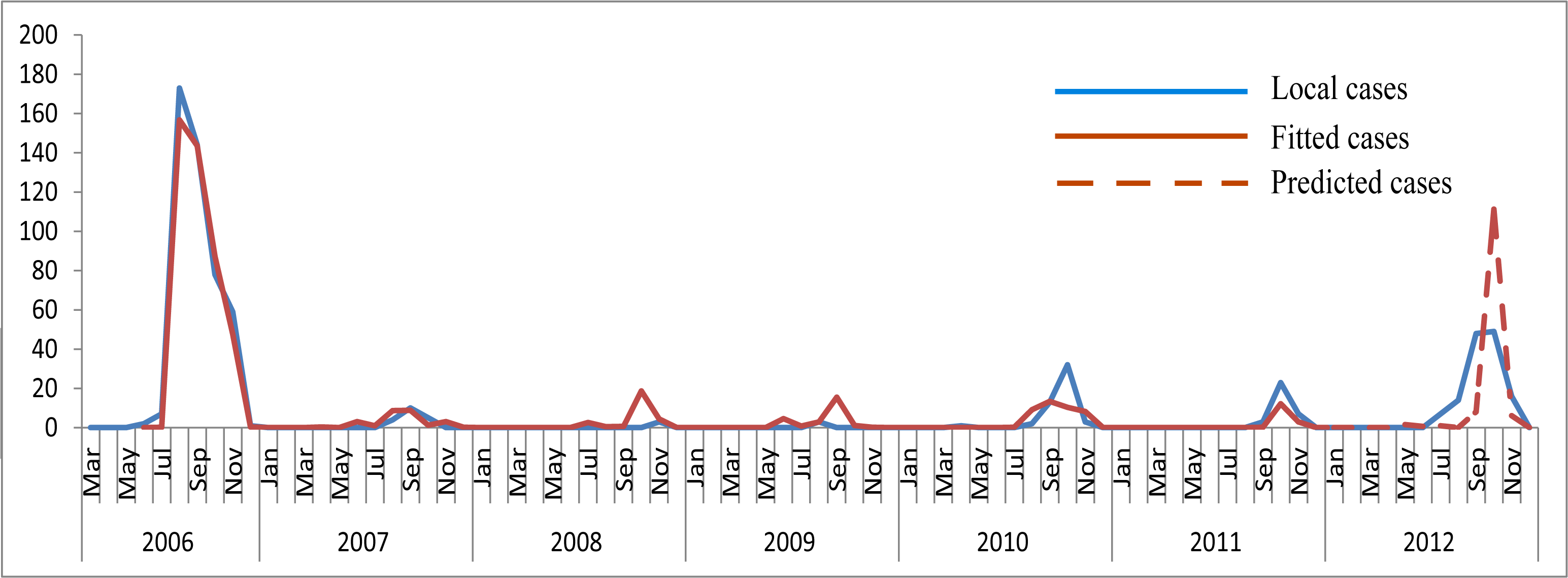

Supplement: Results S1 — The results of predicting model when “Season” variable replaced with three dummy variables (Seasonsummer, Seasonspring and Seasonwinter). (ZIP) [file pone.0102755.s006.zip › Result of dummy variable.tif]
